# Supplementary material for: Status of Asp29 and Asp40 in the Interaction of Naja atra Cardiotoxins with Lipid Bilayers
Source: Toxins (Basel). 2020 Apr 18;12(4):262. doi: 10.3390/toxins12040262 (PMC7232319; doi:10.3390/toxins12040262)
Supplement: Supplementary file 1 [file toxins-12-00262-s001.pdf]

Supplementary Materials: Status of Asp29 and Asp40 in the Interaction of *Naja atra* Cardiotoxins with Lipid Bilayers

Guan-Lin Wu, Yi-Jun Shi, Chia-Hui Huang, Yuan-Chin Lee, Liang-Jun Wang, Jing-Ting Chiou, Chi-Yu Lu and Long-Sen Chang

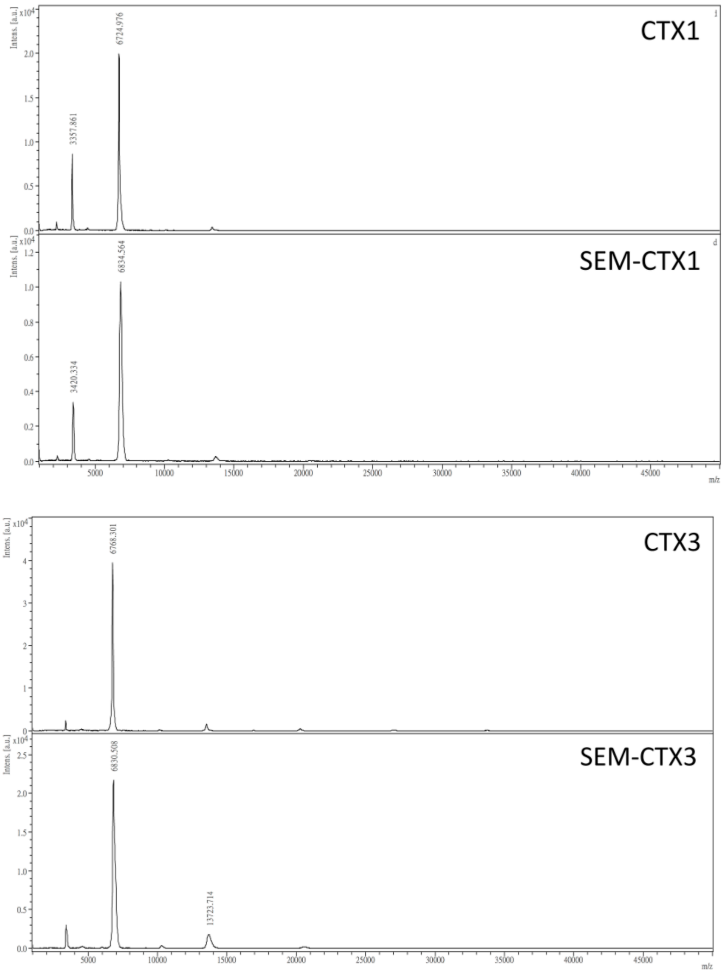

Figure S1. MALDI-TOF mass spectra of CTX1, SEM-CTX1, CTX3, and SEM-CTX3.

Peak 1 YVC(IAA)C(IAA)NTD(SEM)R

#	b	Seq.	y	#
1	164.0706	Y		8
2	263.139	V	981.399	7
3	423.17	C	882.331	6
4	583.2	C	722.3	5
5	697.243	N	562.269	4
6	798.2909	T	448.226	3
7	970.351	D	347.179	2
8		R	175.119	1

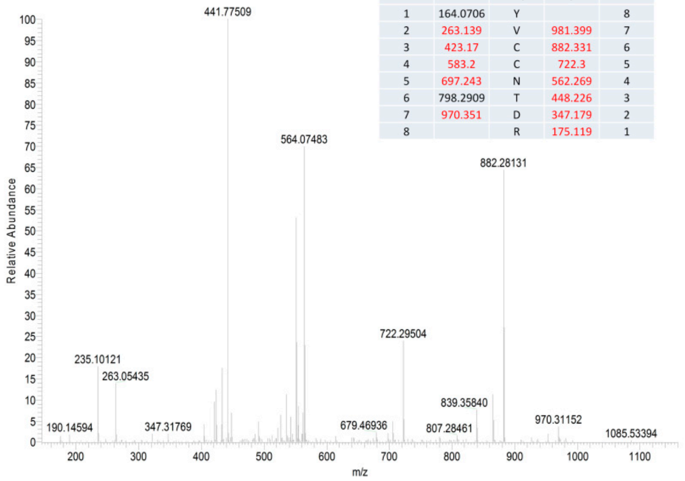

Peak2 RGC(IAA)ID(SEM)VC(IAA)PK

#	b	Seq.	y	#
1	157.1084	R		9
2	214.13	G	1005.46	8
3	374.161	C	948.439	7
4	487.245	I	788.4083	6
5	659.304	D	675.324	5
6	758.3726	V	503.265	4
7	918.4033	C	404.196	3
8	1015.456	P	244.166	2
9		K	147.1128	1

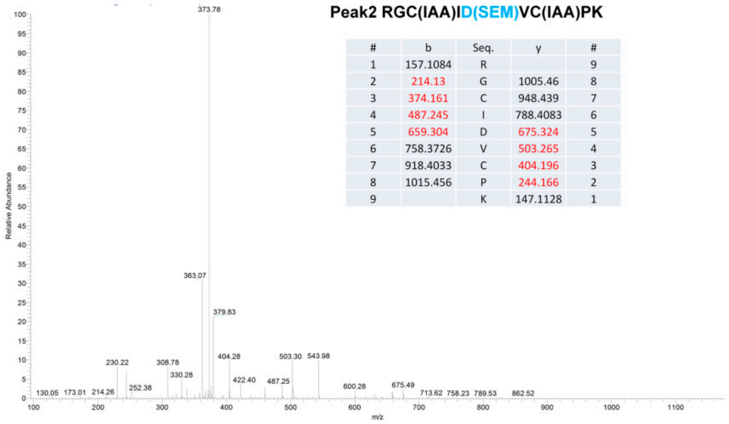

Peak 3 MFMMSD(SEM)LTIPVK

#	b	Seq.	y	#
1	132.0478	M		12
2	279.116	F	1338.69	11
3	410.157	M	1191.62	10
4	541.197	M	1060.582	9
5	628.229	S	929.542	8
6	800.289	D	842.509	7
7	913.373	L	670.45	6
8	1014.421	T	557.366	5
9	1127.5	I	456.318	4
10	1224.557	P	343.234	3
11	1323.63	V	246.181	2
12		K	147.1128	1

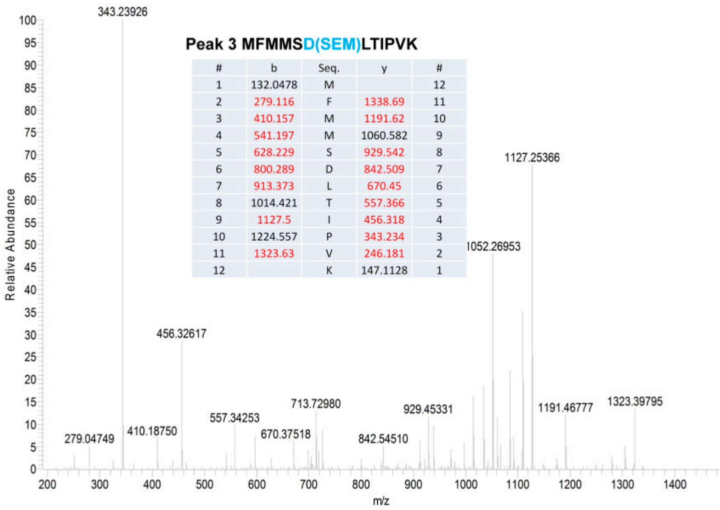

Figure S2. MS/MS analyses for amino acid sequence of semicarbazide-modified peptides derived from the hyrolysates of tryptic RCM-SEM-cariotoxin 1 (CTX1). Peaks 1, 2, and 3 were separated from the hyrolysates of tryptic RCM-SEM-CTX1 (Figure 4). Amino acid sequences of peaks 1, 2, and 3 were determined using NanoUPLC-MS/MS analyses as described in the Materials and Methods section.
